# Patient Questions Surrounding Mask Use for Prevention of COVID-19 and Physician Answers from an Evidence-Based Perspective: a Narrative Review

**DOI:** 10.1007/s11606-020-06324-w

**Published:** 2020-11-03

**Authors:** Jessica A. Martinez, Rachel H. Miller, Ryan A. Martinez

**Affiliations:** 1 The University of Arizona Cancer Center, Tucson, AZ USA; 2 Department of Nutritional Sciences, University of Arizona, Tucson, AZ USA; 3 Tucson Medical Center, Tucson, AZ USA

## Abstract

Recent mandates to wear masks in public places across the USA combined with conflicting messaging from the media and government agencies have generated a lot of patient questions surrounding the appropriate use and efficacy of cloth masks. Here, we have organized the evidence in the context of real patient questions and have provided example answers from a physician’s perspective. The purpose of this review is to offer healthcare providers with examples of how to respond to patient questions about masks in a way that encourages responsible decision-making. We conclude, based on the evidence showing a benefit for cloth masks and the recent reports supporting a role for aerosols in the transmission of SARS-CoV-2, that cloth masks will be effective when used correctly. We further assert that stronger public messaging surrounding cloth masks in the community setting is needed, and should specify that 2–3 layer, fitted face masks be worn at all times in public as another layer of protection in *addition* to social distancing, not just when social distancing cannot be maintained.

## INTRODUCTION

In response to a surge in COVID-19 cases that followed city-wide reopening, the population of Tucson, Arizona, was recently mandated to wear face masks in public places along with several states.^1,2^ Given the conflicting information from the media and government agencies,^3–6^ many patients have questions surrounding the appropriate use and efficacy of cloth masks and are turning to their physicians for clarity and reassurance. To date, there have been no randomized controlled trials designed to test the efficacy of cloth masks in the context of COVID-19, making it difficult for healthcare providers to offer definitive answers. While there have been a plethora of editorials, specials reports, and opinion articles published in the past few months, none, to our knowledge, organizes the evidence in the context of real patient questions. Here, we have summarized ten questions that were submitted to Tucson Medical Center regarding cloth face masks, and have provided example physician responses and scientific rationale. Based on the available evidence—with emphasis on the newest data supporting aerosol transmission of SARS-CoV-2—we believe messaging surrounding cloth masks should promote use in public at all times, in *addition* to social distancing, not just when social distancing cannot be maintained. Clear messaging is particularly important for when individuals are gathered in an enclosed space.

**Question 1**: I am not sick, why do I need to wear a mask?

**Answer**: More than one-third of infected individuals are unaware that they are carriers of the virus and never go on to develop symptoms that warrant testing. By wearing a mask, you protect others. Also, if a person has a recent exposure to someone that has tested positive, but has no symptoms, they should self-isolate as they may be presymptomatic and could spread the virus.

**Rationale**: The CDC estimates that about 40% of infected individuals do not display overt symptoms and may contribute to the significant spread of the disease without their knowledge.^7^ The WHO has stated that patients have detectible levels of SARS-CoV-2 RNA 1–3 days before onset of symptoms.^4^ Using throat swabs to quantify viral loads and information about symptom onset from transmission pairs (*N* = 77 pairs), He et al. estimated that peak infectiousness is 1–2 days prior to onset of symptoms. He further estimated that individuals shed virus 5 to 6 days prior to symptom onset and that total presymptomatic transmission was 44% (CI, 30–57%).^8^ A review of the modeling evidence estimates that asymptomatic and/or presymptomatic spread ranges from 13 to 80%.^9^ Another contributor to asymptomatic/presymptomatic transmission is the high rate of false-negative testing. Using reverse transcriptase polymerase chain reaction (RT-PCR), Kucirka et al. showed that, before onset of symptoms, false-negative rates were 67%.^10^ This evidence taken together suggests that there is a high likelihood that a patient will test negative during their timeframe of peak infectiousness. The most accurate testing was 8 days after a known infection, when many are no longer contagious, and the false-negative rate was still 20%.^10^

**Question 2**: I’ve read that masks do not work.^11^ If some studies caution against cloth masks for healthcare workers,^12^ why should the public use them?

**Answer**: Cloth masks protect other people from you, by reducing the number of droplets that you might release into a shared air space when you breathe, talk, cough, or sneeze. They provide another layer of protection in addition to social distancing and washing your hands. There is evidence to support a benefit for cloth masks in reducing the burden of the COVID-19 pandemic, whereas masks have not been as effective for other respiratory viruses. Medical/surgical masks have a different role. The construction and proper use of a medical mask can help protect the provider from their patients more effectively than a cloth mask such as when exposed to bodily fluids in addition to protecting their patients from respiratory particles.

**Rationale**: The study in question^12^ did show that there were significantly higher rates of respiratory infections (clinical respiratory illness, influenza-like illness, and laboratory-confirmed respiratory virus infection) among practitioners randomized to a cloth mask arm compared to medical grade masks in a large study of practitioners (*N* = 1607). However, the control arm in this study was “usual practice” which included medical mask wearing. Therefore, no conclusions can be drawn as to whether or not a cloth mask provides any level of protection (or not) compared to no mask.

The effectiveness of masks in both community and healthcare settings has been extensively reviewed by Chou et al.^13^; they conclude that, while the body of evidence is not enough to draw firm conclusions, they do point to a benefit for reduced transmission of SARS-CoV-1 or MERS-CoV-1, but no effect for influenza, influenza-like illness, or other viral respiratory illnesses. There have been no studies for SARS-CoV-2 except for observational studies that have shown a *decrease* in cases following implementation of required masking.^14^ A lack of benefit with masking for influenza and the common cold is in agreement with the many commentaries that argue against mask use (e.g., ref.^12^); however, these commentaries fail to reference any studies of viruses more closely related to SARS-CoV-2.

**Question 3**: If I’m standing 6 ft. away from someone while inside, do I need to wear a mask?

**Answer**: Yes, ideally you should wear a mask even if you are able to maintain a distance of 6 ft. The 6 ft. distance is a “best estimate” for how far respiratory droplets spread. There is evidence for spread through aerosols which can travel much farther than droplets, and remain airborne for a longer period of time, and can accumulate in an enclosed space. Social distancing and handwashing do not protect against airborne transmission, masks do.

**Rationale**: It has been demonstrated that pathogen-bearing respiratory particles from exhalations, coughs, and sneezes can travel up to 27 ft. in “turbulent gas clouds” (summarized in^15^). A recent article published in the Physics of Fluids showed that respiratory particles from an emulated cough traveled up to 12 ft. in just 50 s. A folded handkerchief reduced the jet to ~ 3 ft. and a home-made stitched 2-layer cloth mask reduced the forward distance of the jet to less than 3 in.^16^ The WHO and CDC define respiratory particles greater than 5 μm as droplets, and less than 5 μm as aerosols, while others define aerosols as 10 or 20 μm based on the ability of these size respiratory particles to remain airborne (carried within “turbulent gas clouds”) whereas larger droplets typically fall to the ground within the 6 ft. range.^17^ Furthermore, SARS-CoV-2 has been found to remain viable and infectious in aerosols for 3 h.^18^ It is believed that “a few hundred” SARS-CoV-2 particles (virion) are enough to cause disease.^17^ Stadnytskyi estimated that 1 min of loud speaking generates at least 1000 virion-containing aerosols that remain airborne for more than 8 min and that the probability of a given droplet containing 1 SARS-CoV-2 particle is 37%; thus, 1 min of loud speaking may be enough to cause a viral infection beyond the 6-ft. distance.^19^ A recent systematic review of 9 observational studies determined a 8–12% chance of viral infection at 1 m (3.3 ft.), and for each 1-m farther away risk is cut in half.^20^ However, this is environment dependent. In an indoor space, it is likely that aerosols accumulate and travel farther than 6 ft.; this is supported by the documented cluster outbreaks in indoor spaces (summarized by WHO^4^ and CDC^21^).

**Question 4**: What can I use as a filter for my mask and should I try to get filters?

**Answer**: Wearing a mask that has 2–3 layers and is without gaps in conjunction with maintaining distance and hand washing will optimize breathability while still offering enough filtering performance to reduce risk of transmission without the need for an additional filter.

**Rationale**: Layering of textiles improves filtration. In a classic example from public health, it was demonstrated that a folded sari filtered drinking water enough to reduce the incidence of cholera in a village in Bangladesh by 48%.^22^ Emerging epidemiological evidence also supports reduction in risk with face mask use for respiratory infections.^20^ Studies done in the experimental setting have demonstrated excellent filtration of droplets when textiles are layered. For example, Rodriguez-Palacios et al. mimicked a sneeze (180 cm (6 ft.) spray radius) and the number of respiratory particles that passed through various household textiles commonly used for masks. They found that a double layer reduced the number of droplets that passed through by 97.2%. Droplets were prevented from reaching beyond a 30-cm radius with 100% cotton or polyester to < 10 cm radius (similar to a medical mask). Furthermore, with a double layer of 100% cotton or polyester, the circumferential area of contamination was reduced by 99.7%.^23^ Konda et al. reported similar aerosol filtering ability with layers of textiles but noted that mask performance is decreased when gaps are present (such as with bandana use instead of a fitted mask).^24^ Given that the diameter of SARS-CoV-2 is only 100 nm,^25^ a potential line of reasoning against cloth masks is that the virus is too small to be blocked by a textile mask. In an experimental setting, Rengasamy et al. determined that, for 100 nm particles, aerosol penetration of 3 different cloth mask types was 80% (for comparison, penetration of an N95 mask was < 1%). While 80% penetration seems high, the authors note that this is comparable to medical/surgical masks which have a penetration of 51–89%.^26^ Importantly, this study was not conducted using droplets as a carrier for these particles. Respiratory particles (droplets and aerosols) generated from a cough or sneeze are 0.1–900 μm in size (0.1 μm = 100 nm)^27^ and aerosols produced by normal talking or breathing are < 1 μm.^28^ Aydn et al. tested the ability of common household fabrics to block 100 nm particles carried in low- and high-velocity droplets (similar speed to talking and a cough or sneeze respectively); they found that a single layer of 100% cotton blocked as low as 70% of nanoparticles contained in high-velocity droplets, whereas a double layer blocked over 94%, and 3 layers blocked > 98%, which has similar performance to a medical mask. Similarly, 94% of low-velocity droplets were blocked with either single or double layers of 100% cotton. Taken together, this evidence suggests that a layered, fitted mask can perform well enough to reduce disease spread in the community setting without the need for an additional filter.

**Question 5**: Do I have to wear a mask when outside walking?

**Answer**: The Tucson mandate states that people exercising outdoors are not required to wear a mask.^2^ This is supported by studies that show the aerosolized virus is quickly diluted outdoors even though it can remain airborne. Depending on where you are walking, you may still want to wear a mask to reduce the risk to others further.

**Rationale**: In an outdoor space, aerosols are quickly diluted, thus reducing exposure during outdoor activity and making it unlikely to come into contact with a high enough viral load to cause disease (Reviewed by Jayaweera et al.^17^). However, it is important to note that, in areas of high pollution, outdoor virus accumulation can still occur.^14^

**Question 6**: Isn’t it unhealthy to wear a mask? Are not I just breathing in air that is higher in carbon dioxide and depriving myself of oxygen?

**Answer**: The human body is remarkable at maintaining homeostasis. For instance, while exercising you will breathe faster to increase oxygen intake and expel carbon dioxide. Your kidneys also process carbon dioxide. Wearing a mask is not likely to change oxygen or carbon dioxide levels, but it is reassuring to know our bodies can deal with carbon dioxide if needed. For example, surgeons, nurses, and other medical staff wear masks for procedures that are 8+ h long and are able to practice for many years without harmful effects from carbon dioxide.

**Rationale**: There are currently no published clinical trials to address whether or not cloth masks increase risk for hypercapnea, for normal, healthy adults. The kinetic diameter of CO_2_ is 330 picometer (3.3 × 10^−6^ μm),^29^ much smaller than the weave of cloth masks; thus, hypercapnea with cloth mask use is extremely unlikely. Similarly, the kinetic diameter of oxygen is 346 picometers;^30^ thus, the exchange of CO_2_ and O_2_ is not affected by the use of cloth masks. There is a potential concern for individuals with respiratory diseases like asthma; however, in agreement with the WHO and CDC, the Asthma and Allergy Foundation of America recommends that people with asthma wear a face-covering unless they have trouble breathing.^31^

**Question 7**: Why would someone wear a mask in their car, when they are alone?

**Answer**: Before you laugh at the solo occupant of a car wearing a mask, consider they may be traveling between errands, or between appointments. Not touching or frequently adjusting the mask means less chance of transferring the virus via contact if present. Before you put on or remove your mask, you should wash your hands properly with soap and water for 20 s.

**Rationale**: SARS-CoV-2 has been shown to be viable on surfaces for days (although the half-life is a few hours) depending on the surface.^18^ One study has demonstrated that, after 24 h on a cotton surface, 99.995% of SARS-CoV-2 was lost, and there was no detectible virus after 24 h. Conversely, SARS-CoV-2 lived for 14 days on the surface of an N95 respirator (preprint article^32^).

**Question 8**: How often should I change and clean my mask? Can I take my mask on and off throughout the day and it still be effective?

**Answer**: You should use a clean cloth mask every day, or if your mask gets dirty or damp. Masks can be washed in the regular laundry or with a bleach solution—make sure the bleach is intended for disinfection as color-safe bleach for clothing is not for disinfecting. If you need to remove your mask throughout the day, you need to follow proper technique for removing and reapplying your mask, along with proper hand hygiene. The CDC recommends that, to remove your mask, only touch the ear loops or ties, then fold the corners together so that you only touch the inside of your mask. The more you touch and adjust a mask, the more chances of contamination (see Question 7).

**Rationale**: Regular soap is an effective means to deactivate multiple virus types including SARS-CoV-2.^33^ The CDC provides instructions on how to wash your cloth face mask in the washing machine or by hand.^34^ Briefly, in the washing machine, masks should be washed with detergent on the warmest water setting possible. If washing by hand, the CDC recommends using bleach intended for disinfection and contains 5.25–8.25% sodium hypochlorite. Mix 5 tablespoons bleach per gallon of water and let the mask soak for 5 min. Make sure the mask is completely dry before use.

**Question 9**: Will ultraviolet rays (UV) from the sunlight kill the virus?

**Answer**: No, at least not to any significant amount. Only UVA and UVB reach the earth and have not shown any effect on SARS-CoV-2; though UVC has been shown to deactivate SARS-CoV-2 in some studies, it is absorbed by the ozone layer so it does not reach the earth.

**Rationale**: A study in China showed that high temperature and UV radiation did not reduce the transmission of SARS-CoV-2.^35^ Conversely, another large study showed an association with higher UV exposure and a lower number of COVID-19 cases (*N* = 10,000).^36^ However, the study was conducted only in January and February; thus, the UV index was very low for the majority of locations, with less than 5 (of 85) locations reaching a UV index of 7 or higher in either month; until yearly data is available, these results should be considered inconclusive. In tightly controlled laboratory conditions, only UVC, which is completely absorbed by the ozone layer,^37^ has been shown to disinfect SARS-CoV-2^38^ and SARS-CoV-1.^39^ Anecdotally, for much of the summer it was over 100 °F most days in Tucson with a UV index of about 9, and the number of new cases each day continued to increase until the masking order was put into effect.

**Question 10**: If I’m in my own office in a building with shared air ventilation, do I need to wear a mask?

**Answer**: No. The amount of droplets containing virus particles would likely not be concentrated enough to cause disease. However, please follow your company’s policies regarding mask wearing.

**Rationale**: Some studies have found the presence of SARS-CoV-2 RNA in air samples, including in the exhaust vent^40,41^; however, these studies were not quantitative and were unable to determine viability of the virus. Other studies were not able to detect airborne viral RNA.^42,43^ A recent commentary summarized the evidence for airborne transmission of SARS-CoV-2, noting that risk for airborne transmission is highest in indoor spaces with poor ventilation.^44^ Recently, Mouchtouri et al. identified positive SARS-CoV-2 samples in the air as well as the central air outlet on the roof of the hospital that was connected to the patient’s room, suggesting that SARS-CoV-2 traveled through the ventilation system.^45^ However, ventilation was connected to a negative pressure room, and the authors were not able to quantify virus levels or determine virus viability.

## CONCLUSION

Here, we have offered providers with examples of how to respond to patient questions about masks in a way that encourages prudence without propagating fear. Given that there is no evidence that cloth masks cause harm for healthy adults, they can be another layer of protection to be used in conjunction with social distancing and frequent hand washing. Areas that have heeded such advice and implemented mask use have seen a decline in new daily cases, whereas areas with social distancing measures alone have not.^14^ Some of the messaging surrounding masks states that masks should be worn in “specific situations” and *when social distancing is not possible.*^4,46^ We assert that stronger public messaging is necessary, and should specify that 2–3 layer, fitted face masks should be worn at all times in public as another layer of protection even when social distancing is possible. Opponents of face masks argue that there is no evidence of effect for masks. As Leung wisely stated in March, “Absence of evidence of effectiveness is not the same as evidence of ineffectiveness, especially when facing a novel situation with limited alternative options”.^47^ We, the authors, believe masks will be effective when used correctly. At the very least, cloth masks can serve as a reminder that, while some shut-downs have eased, COVID-19 has not.

## References

[1] **CNN news**. These are the states requiring people to wear masks when out in public 2020 [updated July 6; cited 2020 July 11]. Available from: https://www.cnn.com/2020/06/19/us/states-face-mask-coronavirus-trnd/index.html.

[2] **Romero MR**. Proclamation of the Mayor declaring a continuing emergency or local emergency related to COVID-19 and declaring regulations and advisories necessary for public safety and protection of life and to mitigate the spread of COVID-19 2020 [updated 06/18/2020; cited 2020 06/30/2020]. Available from: https://bloximages.chicago2.vip.townnews.com/tucson.com/content/tncms/assets/v3/editorial/7/93/793ecd7e-b1ae-11ea-a1be-87229c8b8c1f/5eebe38ba3496.pdf.pdf.

[3] **World Health Organization**. Coronavirus disease (COVID-19) advice for the public 2020 [updated June 4; cited 2020 June 23]. Available from: https://www.who.int/emergencies/diseases/novel-coronavirus-2019/advice-for-public.

[4] **World Health Organizaton**. Transmission of SARS-CoV-2: implications for infection prevention precautions 2020 [updated July 9; cited 2020 July 9]. Available from: https://www.who.int/news-room/commentaries/detail/transmission-of-sars-cov-2-implications-for-infection-prevention-precautions.

[5] Feng S, Shen C, Xia N, Song W, Fan M, Cowling BJ (2020). Rational use of face masks in the COVID-19 pandemic. Lancet Respir Med.

[6] **Centers for Disease Control and Prevention**. Use of Cloth Face Coverings to Help Slow the Spread of COVID-19 2020 [updated June 28; cited 2020 July 11]. Available from: https://www.cdc.gov/coronavirus/2019-ncov/prevent-getting-sick/diy-cloth-face-coverings.html.

[7] **Centers for Disease Control**. COVID-19 pandemic planning scenarios 2020 [updated July 10; cited 2020 Aug 31]. Available from: https://www.cdc.gov/coronavirus/2019-ncov/hcp/planning-scenarios.html.

[8] He X, Lau EHY, Wu P, Deng X, Wang J, Hao X (2020). Temporal dynamics in viral shedding and transmissibility of COVID-19. Nat Med..

[9] **Furukawa NW, Brooks JT, Sobel J**. Evidence supporting transmission of severe acute respiratory syndrome coronavirus 2 while presymptomatic or asymptomatic. Emerg Infect Dis. 2020;26(7). 10.3201/eid2607.201595. 10.3201/eid2607.201595 PMC7323549 32364890

[10] **Kucirka LM, Lauer SA, Laeyendecker O, Boon D, Lessler J**. Variation in false-negative rate of reverse transcriptase polymerase chain reaction-based SARS-CoV-2 tests by time since exposure. Ann Intern Med. 2020. 10.7326/M20-1495. 10.7326/M20-1495 PMC7240870 32422057

[11] **Rancourt DG**. Masks Don’t Work: A Review of Science Relevant to COVID-19 Social Policy 2020 [updated June 11, 2020; cited 2020 July 9, 2020]. Available from: https://www.rcreader.com/commentary/masks-dont-work-covid-a-review-of-science-relevant-to-covide-19-social-policy?fbclid=IwAR1iezfxlpuJneVh6AgFlpZND3-2-qlaZO53vXF6UHO-KRKxvFupTbgOrdk.

[12] MacIntyre CR, Seale H, Dung TC, Hien NT, Nga PT, Chughtai AA (2015). A cluster randomised trial of cloth masks compared with medical masks in healthcare workers. BMJ Open..

[13] **Chou R, Dana T, Jungbauer R, Weeks C, McDonagh MS**. Masks for prevention of respiratory virus infections, including SARS-CoV-2, in health care and community settings: a living rapid review. Ann Intern Med. 2020. 10.7326/M20-3213. 10.7326/M20-3213 PMC7322812 32579379

[14] Zhang R, Li Y, Zhang AL, Wang Y, Molina MJ (2020). Identifying airborne transmission as the dominant route for the spread of COVID-19. Proc Natl Acad Sci U S A..

[15] **Bourouiba L.** Turbulent gas clouds and respiratory pathogen emissions: potential implications for reducing transmission of COVID-19. JAMA. 2020. 10.1001/jama.2020.4756. 10.1001/jama.2020.4756 32215590

[16] Verma S, Dhanak M, Frankenfield J (2020). Visualizing the effectiveness of face masks in obstructing respiratory jets. Physics of Fluids..

[17] Jayaweera M, Perera H, Gunawardana B, Manatunge J (2020). Transmission of COVID-19 virus by droplets and aerosols: a critical review on the unresolved dichotomy. Environ Res..

[18] van Doremalen N, Bushmaker T, Morris DH, Holbrook MG, Gamble A, Williamson BN (2020). Aerosol and surface stability of SARS-CoV-2 as compared with SARS-CoV-1. N Engl J Med..

[19] Stadnytskyi V, Bax CE, Bax A, Anfinrud P (2020). The airborne lifetime of small speech droplets and their potential importance in SARS-CoV-2 transmission. Proc Natl Acad Sci U S A..

[20] **Chu DK, Akl EA, Duda S, Solo K, Yaacoub S, Schunemann HJ, et al.** Physical distancing, face masks, and eye protection to prevent person-to-person transmission of SARS-CoV-2 and COVID-19: a systematic review and meta-analysis. Lancet. 2020. 10.1016/S0140-6736(20)31142-9. 10.1016/S0140-6736(20)31142-9 PMC7263814 32497510

[21] **Furuse Y, Sando E, Tsuchiya N, Miyahara R, Yasuda I, Ko YK, et al.** Clusters of coronavirus disease in communities, Japan, January-April 2020. Emerg Infect Dis. 2020;26(9). 10.3201/eid2609.202272. 10.3201/eid2609.202272 PMC7454082 32521222

[22] **Huq A, Yunus M, Sohel SS, Bhuiya A, Emch M, Luby SP, et al.** Simple sari cloth filtration of water is sustainable and continues to protect villagers from cholera in Matlab, Bangladesh. mBio. 2010;1(1). 10.1128/mBio.00034-10. 10.1128/mBio.00034-10 PMC2912662 20689750

[23] Rodriguez-Palacios A, Cominelli F, Basson AR, Pizarro TT, Ilic S (2020). Textile masks and surface covers-a spray simulation method and a “universal droplet reduction model” against respiratory pandemics. Front Med (Lausanne)..

[24] Konda A, Prakash A, Moss GA, Schmoldt M, Grant GD, Guha S (2020). Aerosol filtration efficiency of common fabrics used in respiratory cloth masks. ACS Nano.

[25] **Bar-On YM, Flamholz A, Phillips R, Milo R**. SARS-CoV-2 (COVID-19) by the numbers. Elife. 2020;9. 10.7554/eLife.57309. 10.7554/eLife.57309 PMC7224694 32228860

[26] Rengasamy S, Eimer B, Shaffer RE (2010). Simple respiratory protection--evaluation of the filtration performance of cloth masks and common fabric materials against 20-1000 nm size particles. Ann Occup Hyg..

[27] Zayas G, Chiang MC, Wong E, MacDonald F, Lange CF, Senthilselvan A (2012). Cough aerosol in healthy participants: fundamental knowledge to optimize droplet-spread infectious respiratory disease management. BMC Pulm Med.

[28] Anderson EL, Turnham P, Griffin JR, Clarke CC (2020). Consideration of the aerosol transmission for COVID-19 and public health. Risk Anal.

[29] Scholes CA, Kentish SE, Stevens GW (2008). Carbon dioxide separation through polymeric membrane systems for flue gas applications. Recent Patents on Chemical Engineering.

[30] Mehio N, Dai S, Jiang DE (2014). Quantum mechanical basis for kinetic diameters of small gaseous molecules. J Phys Chem A..

[31] **Asthma and Allergy Foundation of America**. What People With Asthma Need to Know About Face Masks and Coverings During the COVID-19 Pandemic 2020 [cited 2020 July 11]. Available from: https://community.aafa.org/blog/what-people-with-asthma-need-to-know-about-face-masks-and-coverings-during-the-covid-19-pandemic.

[32] **Kasloff SB, Strong JE, Funk D, Cutts TA**. Stability of SARS-CoV-2 on critical personal protective equipment. medRxiv [Preprint]. 2020. 10.1101/2020.06.11.20128884 10.1038/s41598-020-80098-3 PMC7806900 33441775

[33] **Ma QX, Shan H, Zhang HL, Li GM, Yang RM, Chen JM**. Potential utilities of mask-wearing and instant hand hygiene for fighting SARS-CoV-2. J Med Virol. 2020. 10.1002/jmv.25805. 10.1002/jmv.25805 PMC7228401 32232986

[34] **Centers for Disease Control and Prevetion**. How to Wash Cloth Face Coverings 2020 [updated May 22; cited 2020 July 11]. Available from: https://www.cdc.gov/coronavirus/2019-ncov/prevent-getting-sick/how-to-wash-cloth-face-coverings.html.

[35] **Yao Y, Pan J, Liu Z, Meng X, Wang W, Kan H, et al.** No association of COVID-19 transmission with temperature or UV radiation in Chinese cities. Eur Respir J. 2020;55(5). 10.1183/13993003.00517-2020. 10.1183/13993003.00517-2020 PMC7144256 32269084

[36] **Gunthe SS, Swain B, Patra SS, Amte A**. On the global trends and spread of the COVID-19 outbreak: preliminary assessment of the potential relation between location-specific temperature and UV index. Z Gesundh Wiss. 2020:1-10. 10.1007/s10389-020-01279-y. 10.1007/s10389-020-01279-y PMC7180684 32337151

[37] Maverakis E, Miyamura Y, Bowen MP, Correa G, Ono Y, Goodarzi H (2010). Light, including ultraviolet. J Autoimmun..

[38] **Ozog DM, Sexton JZ, Narla S, Pretto-Kernahan CD, Mirabelli C, Lim HW, et al.** The effect of ultraviolet c radiation against SARS-CoV-2 inoculated N95 respirators. [Journal Article]. In press 2020. 10.1016/j.ijid.2020.08.077 PMC7470719 32891736

[39] **Aboubakr HA, Sharafeldin TA, Goyal SM**. Stability of SARS-CoV-2 and other coronaviruses in the environment and on common touch surfaces and the influence of climatic conditions: a review. Transbound Emerg Dis. 2020. 10.1111/tbed.13707. 10.1111/tbed.13707 PMC7361302 32603505

[40] Guo ZD, Wang ZY, Zhang SF, Li X, Li L, Li C (2020). Aerosol and surface distribution of severe acute respiratory syndrome coronavirus 2 in hospital wards, Wuhan, China, 2020. Emerg Infect Dis..

[41] Chia PY, Coleman KK, Tan YK, Ong SWX, Gum M, Lau SK (2020). Detection of air and surface contamination by SARS-CoV-2 in hospital rooms of infected patients. Nat Commun.

[42] Faridi S, Niazi S, Sadeghi K, Naddafi K, Yavarian J, Shamsipour M (2020). A field indoor air measurement of SARS-CoV-2 in the patient rooms of the largest hospital in Iran. Sci Total Environ.

[43] **Ong SWX, Tan YK, Chia PY, Lee TH, Ng OT, Wong MSY, et al.** Air, surface environmental, and personal protective equipment contamination by severe acute respiratory syndrome coronavirus 2 (SARS-CoV-2) from a symptomatic patient. JAMA. 2020. 10.1001/jama.2020.3227. 10.1001/jama.2020.3227 PMC7057172 32129805

[44] **Morawska L, Tang JW, Bahnfleth W, Bluyssen PM, Boerstra A, Buonanno G, et al.** How can airborne transmission of COVID-19 indoors be minimised? Environ Int. 2020;142:105832. 10.1016/j.envint.2020.105832. 10.1016/j.envint.2020.105832 PMC7250761 32521345

[45] Mouchtouri VA, Koureas M, Kyritsi M, Vontas A, Kourentis L, Sapounas S (2020). Environmental contamination of SARS-CoV-2 on surfaces, air-conditioner and ventilation systems. Int J Hyg Environ Health.

[46] **World Health Organization**. Coronavirus disease (COVID-19) advice for the public: When and how to use masks 2020 [updated June 19; cited 2020 July 6]. Available from: https://www.who.int/emergencies/diseases/novel-coronavirus-2019/advice-for-public/when-and-how-to-use-masks.

[47] Leung CC, Lam TH, Cheng KK (2020). Mass masking in the COVID-19 epidemic: people need guidance. Lancet.

